# Survival benefits of para-aortic lymphadenectomy in colorectal cancer with clinically suspected para-aortic lymph node metastasis: a meta-analysis and systematic review

**DOI:** 10.1186/s12957-023-02908-y

**Published:** 2023-01-31

**Authors:** Rong-Chang Wang, Jian-Qi Wang, Xiao-Yu Zhou, Chu-lin Zhong, Jin-Xu Chen, Jing-Song Chen

**Affiliations:** Department of Gastrointestinal Surgery, The First Affiliated Hospital of Guangzhou Medical University, Guangzhou, 510120 People’s Republic of China

**Keywords:** Colorectal cancer, Para-aortic lymph node, Para-aortic lymphadenectomy, Survival, Metastasis

## Abstract

**Background and objectives:**

In patients with colorectal cancer and clinically suspected para-aortic lymph node metastasis, the survival benefit of para-aortic lymphadenectomy is unknown. We conducted a meta-analysis and systematic review to investigate it.

**Methods:**

PubMed, Web of Science, and EMBASE were searched until January 2000 to April 2022 to identify studies reporting overall survivals, complication rates, and hazard ratios of prognostic factors in patients with colorectal cancer undergoing para-aortic lymphadenectomy, and those data were pooled.

**Results:**

Twenty retrospective studies (1021 patients undergoing para-aortic lymphadenectomy) met the inclusion criteria. Meta-analysis indicates that participants undergoing para-aortic lymphadenectomy were associated with 5-year survival benefit, compared to those not receiving para-aortic lymphadenectomy (odds ratio = 3.73, 95% confidence interval: 2.05–6.78), but there was no significant difference in complication rate (odds ratio = 0.97, 95% confidence interval: 0.46–2.08). Further analysis of para-aortic lymphadenectomy group showed that 5-year survival of the positive group with pathologically para-aortic lymph node metastasis was lower than that of the negative group (odds ratio = 0.19, 95% confidence interval: 0.11–0.31). Moreover, complete resection (odds ratio = 5.26, 95% confidence interval: 2.02–13.69), para-aortic lymph node metastasis (≤4) (hazard ratio = 1.88, 95% confidence interval: 0.97–3.62), and medium-high differentiation (hazard ratio = 2.98, 95% confidence interval: 1.48–5.99) were protective factors for survival. Preoperative extra-retroperitoneal metastasis was associated with poorer relapse-free survival (hazard ratio = 1.85, 95% confidence interval: 1.10–3.10).

**Conclusion:**

Para-aortic lymphadenectomy had promising clinical efficacy in prolonging survival rather than complication rate in patients with colorectal cancer and clinically diagnostic para-aortic lymph node metastasis. Further prospective studies should be performed.

**Trial registration:**

PROSPERO: CRD42022379276.

**Supplementary Information:**

The online version contains supplementary material available at 10.1186/s12957-023-02908-y.

## Introduction

Colorectal cancer (CRC) is the third most commonly diagnosed cancer and is the second-leading cause of cancer-related deaths worldwide, with an incidence of over 1.9 million new cases and more than 935,000 deaths in 2020 according to the GLOBOCAN 2020 report [1]. Compared to liver and lung metastasis, para-aortic lymph node (PALN) metastasis is a particularly rare pattern of distant metastasis with an occurrence rate of less than 2% [2]. Once PALN metastasis occurs, it may lead to an early recurrence after surgery and extremely worse survival in patients with pancreatic cancer, biliary cancer, or cervix cancer [3–5]. Surgical excision of primary and metastatic lesions is still considered to be the most effective way to cure CRC with distant metastasis. Imaging data are reproducible and effective methods for routine clinical diagnosis of PALN metastasis [6]. However, the best treatment for patients with CRC and clinically suspected PALN metastasis is still controversial due to the different definitions in the past.

The 2017 AJCC 8^th^ Edition TNM staging classified PALN metastasis in patients with CRC as stage M1 or distant metastasis, rather than grouping it with regional lymph nodes [6]. PALN metastasis may occur in the form of an oligometastatic state [7], which provides an opportunity for patients to receive radical para-aortic lymphadenectomy (PALND). So far, many studies have pointed out the survival benefit of PALND for clinically diagnosed PALN metastasis in patients with CRC. However, due to the small sample data and no prospective studies, the evidence level is not convincing enough.

The era of precision medicine warrants the application of a more personalized approach for the treatment of patients with CRC and clinically suspected PALN metastasis. Since PALN is close to important blood vessels, surgery is somewhat dangerous and difficult. The question of which type of patients receiving PALND would benefit from survival also needs to be answered. In this study, we aimed to perform a meta-analysis of survival outcomes and prognostic factors in patients with CRC and clinically suspected PALN metastasis undergoing PALND.

## Material and methods

### Search strategy

A literature search was performed on PubMed, Web of Science, and Embase databases for studies on PALN management of CRC published in English between 1 January 2000 and 20 April 2022. Medical search headings, “colorectal cancer,” “para-aortic,” “retroperitoneal lymph nodes,” “recurrence,” “metastasis,” and “lymphadenectomy” were used. All searched citations were imported into EndNote software to eliminate all duplicates. Titles and abstracts of potential studies were scanned to exclude all irrelevant studies. The remaining articles that did not meet the inclusion criteria were removed. The study selection process was summarized in a flow diagram.

### Selection criteria

Inclusion criteria were as follows: articles published in English; studies in humans; patients with CRC; definitive radiographic evidence of PALN metastasis such as computed tomography, magnetic resonance imaging, and positron emission tomography; and studies reporting overall survivals (OSs), complication rates, and hazard ratios (HRs) of prognostic factors in patients with CRC undergoing PALND or those data could be extracted from Kaplan–Meier graphs.

The following were used as exclusion criteria: a repeat trial in the same cohort; another source of cancer; studies that were not focused on the subject of our investigation; full-text studies that could not be obtained; and unaccomplished prospective studies, reviews, comments, meta-analysis, and case studies.

### Date extraction and analysis

After study selection, two investigators (J.-Q.W. and C.-L.Z.) independently collected information from all eligible studies. Any difference was solved by a third author (X.-Y.Z.) with a consultation. Data were collected using Excel form, and the following information was extracted: first author, year of publication, country, sample size, the surgery timeline of primary CRC and suspected metastatic PALN, lymph node dissection information, CR, OS, and HR. The pooled data were selected as OS, HR and its 95% confidence interval (CI), and CR. If the study did not provide the above data, survival data were extracted from the Kaplan–Meier curves by the software Engauge Digitizer version 4.1 [8]. The methodological quality of studies was assessed using the Newcastle-Ottawa Scale (NOS) tool.

Review Manager V.5.4 software and Stata/SE V16.0 for Windows software were used to conduct statistical analysis. *P* value<0.05 was defined as statistically significant. The outcome values were estimated as descriptive statistics and 95% CIs. *I*^2^ statistic was used to check heterogeneity across eligible studies [9]. *I*^2^≤50% implied acceptable homogeneity. A random-effect model was applied to all pooled values due to the inclusion of non-randomized trials and the inevitable existence of heterogeneity [9]. If there was obvious heterogeneity, its sources were scrutinized. Sensitivity analysis and subgroup analysis were used to examine heterogeneous article sources. A funnel plot was used to analyze the reporting bias, and Begg’s test was performed, if possible.

## Results

### Literature search results

**Fig. 1:**
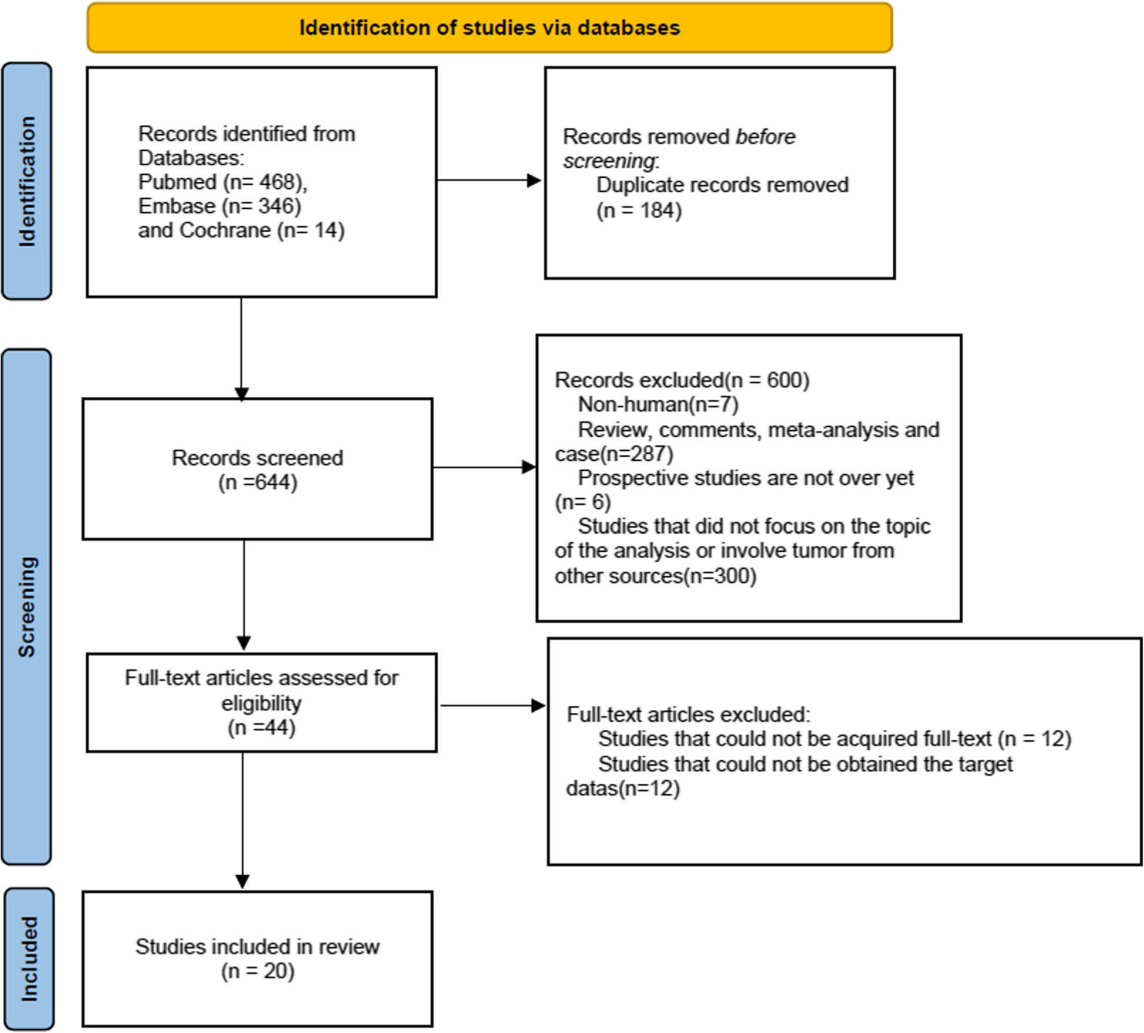
PRISMA 2020 flow diagram for systematic reviews

The search yielded a total of 828 citations comprising 468 publications in PubMed, 346 in Embase, and 14 in Cochrane Central Register between January 2000 and April 2022, 184 duplicates of which were excluded in the first screening. We identified 644 potentially relevant studies that were retrieved and reviewed by titles and abstracts, 44 of which remained, and then 24 were excluded by reading the full text of the original text. Finally, 20 retrospective studies were included in our meta-analysis (Fig. 1).

### Characteristics of included studies

Twenty retrospective trials [2, 7, 10–27] enrolled a total of 1021 patients with CRC and clinically suspected PALN metastasis undergoing PALND, of which 613 patients were pathologically positive for PALN and conversely, 408 patients were pathologically negative for PALN. Twenty articles detailed descriptions of patients with CRC undergoing PLAND, of which 8 reported 339 control patients who did not receive PALND. Based on the surgical timelines of the primary tumor and PALN, the enrolled studies were divided into two groups, one was synchronous surgery and the other was metachronous surgery. The differentiation degree of CRC was as follows: high differentiation, medium differentiation, and poor differentiation. The surgical approaches were open and laparoscopic, and the standard for complete resection (R0) of para-aortic lymphadenectomy was microscopically free of cell residue. All of the studies provided OS, only 3/20 provided the postoperative CRs in the control group, and most (12/20) performed Cox’s proportional hazard models. The mean NOS score of the included literature was 6.55 (Table 1).

**Table 1:** Basic information of eligible studies for para-aortic lymphadenectomy

Author	Year	Country	Sample size	Resection margin	Time to para-aortic lymphadenectomy (synchronous/metachronous)	Number of positive para-aortic lymph nodes	Sample size of control group	Pooled value	NOS score
Min	2008	Korea	6	R0: 6	Metachronous	6	32	OS	6
Lee, S.C.	2021	Korea	47	No mentioned	Synchronous	47	26	OS, HR, CR	7
Choi	2015	Korea	24	No mentioned	Metachronous	24	53	OS, HR, CR	7
Kim	2020	Korea	16	No mentioned	Metachronous	13	19	OS	7
Nozawa	2020	Japan	11	No mentioned	No mentioned	11	130	OS	7
Ogura	2015	Japan	16	R0: 16	Synchronous	10	12	OS, CR	7
Shibata	2002	America	20	R0: 15 Not R0: 5	Metachronous	20	5	OS, HR	6
Tentes	2007	Greece	62	RO: 62	Synchronous	62	62	OS	7
Gagnière	2015	France	25	Not R0: 25	Both (19:6)	25		OS, HR	6
Song	2016	Korea	40	No mentioned	Synchronous	16		OS, HR	6
Sahara	2019	Germany	322	R0: 246 Not R0: 75	Synchronous	62		OS, HR	7
Nakai	2017	Japan	30	R0: 18 Not R0: 12	Synchronous	30		OS	7
Sakamoto	2020	Japan	29	R0:29	Synchronous	29		OS, HR	6
Lee, S.H.	2017	Korea	27	No mentioned	Synchronous	27		OS	7
Bae	2018	Korea	49	No mentioned	Synchronous	49		OS, HR	6
Ichikawa	2021	Japan	28	R0: 17 Not R0: 11	Both (16:12)	28		OS, HR	6
Dumont	2012	France	31	R0: 31	Metachronous	23		OS	7
Razik	2013	Canada	48	R0: 37 Not R0: 11	Metachronous	48		OS, HR	6
Yamada	2018	Japan	36	No mentioned	Synchronous	36		OS, HR	6
Sun	2021	China	154	R0: 145 NotR0: 9	Synchronous	47		OS, HR	7

*R0* microscopically free of cell residue, *OS* overall survival, *HR* hazard ratio, *CR* complication rate

### Survival outcomes

**Fig. 2:**
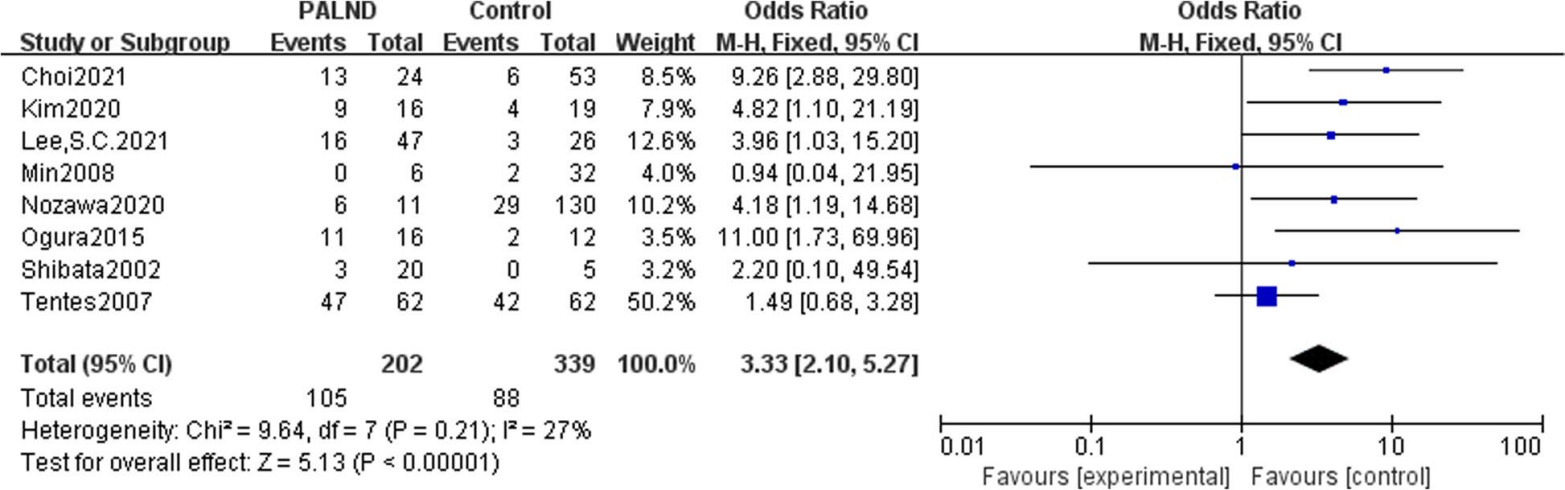
Meta-analysis comparing patients with colorectal cancer and clinically suspected PALN metastasis receiving PALND with those did not undergo PALND and further analysis of the prognostic factors in PALND group. Forest plot of odds ratio for 5-year overall survival between PALND group and control group

**Fig. 3:**
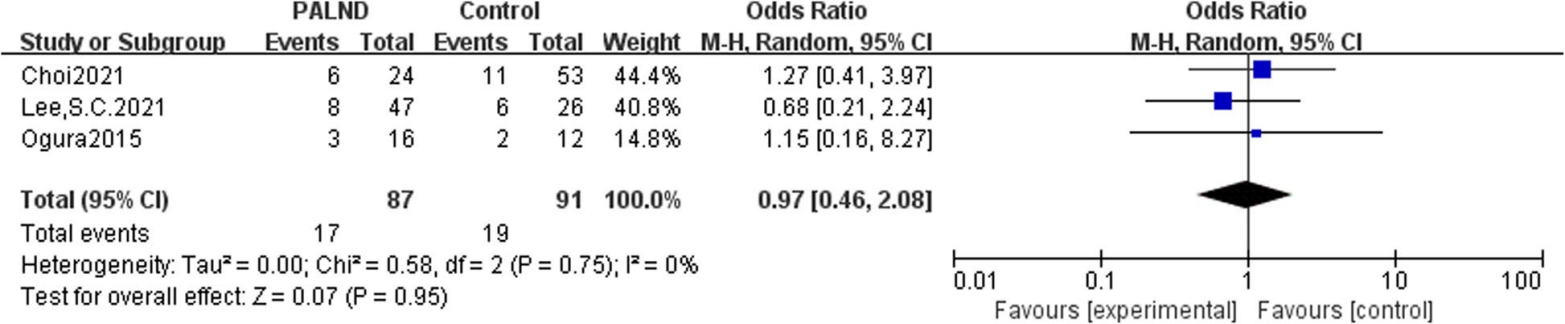
Meta-analysis comparing patients with colorectal cancer and clinically suspected PALN metastasis receiving PALND with those did not undergo PALND, and further analysis of the prognostic factors in PALND group. Forest plot of odds ratio for complication rate between PALND group and control group

In all of the eight studies included, there were no preoperative metastases other than para-aortic lymph nodes. Patients with CRC and clinically suspected PALN metastasis were divided into the experimental group receiving PALND and the control group receiving only adjuvant chemotherapy. Analysis results indicated that patients with CRC and clinically suspected PALN metastasis undergoing PLAND had an advantage 5-year OS than those did not receive PALND, with odds ratio (OR) of 3.73(95% CI: 2.05-6.78) (Fig. 2). Three articles described CRs in the PALND group versus the control group, and meta-analysis showed that there was no difference in the postoperative CRs (OR = 0.97, 95% CI: 0.46–2.08) (Fig. 3). Due to lack of operative data, it is not possible to further analyze the length of hospital stay, amount of blood loss, and recovery time of intestinal peristalsis function.

### Subgroup analysis of main outcome

**Fig. 4:**
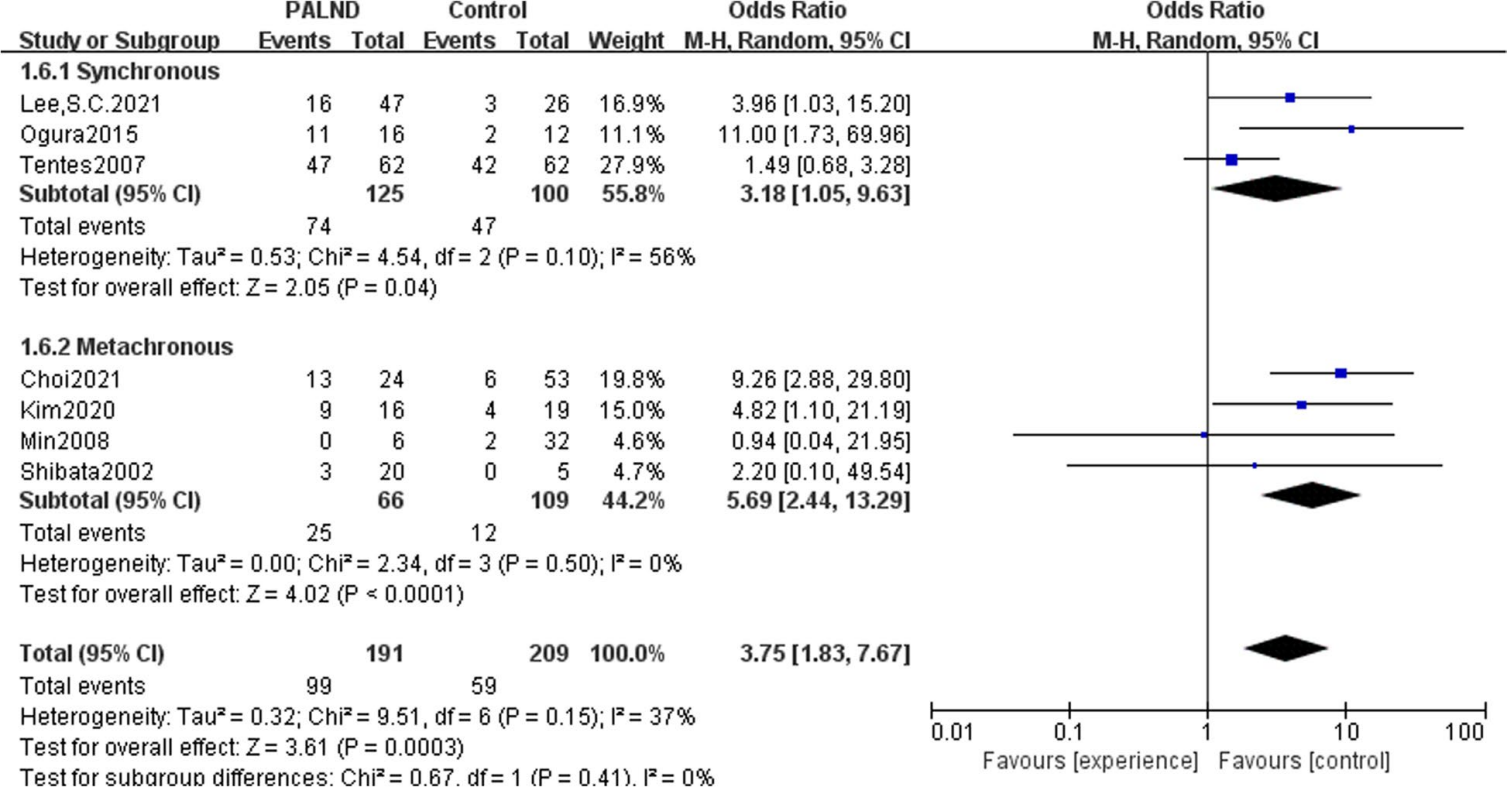
Meta-analysis comparing patients with colorectal cancer and clinically suspected PALN metastasis receiving PALND with those did not undergo PALND, and further analysis of the prognostic factors in PALND group. Forest plot of subgroup analysis for 5-year overall survival between PALND group and control group

In the aforementioned meta-analysis of 5-year OS between the PALND group and the control group, we took into account the time to surgery of primary tumor and PALN, so a subgroup analysis was performed and the results were consistent with the total result that there was a survival benefit for patients who underwent PLAND, both in the synchronous surgery group (OR = 3.18, 95% CI: 1.05-9.63) and in the metachronous group (OR = 5.69, 95% CI: 2.44-13.29) (Fig. 4).

### Further analysis of the population treated with PALND in the twenty studies

The 5-year OS of the pathologically PALN-positive group was worse than that of the pathologically PALN-negative group (OR = 0.19, 95% CI: 0.11-0.31). Similarly, the results were consistent when comparing 3-year OS (OR = 0.30, 95% CI: 0.11–0.82). Additionally, we found that patients undergoing PALND had a better prognosis in the R0 group than in the non-complete resection (non-R0) group (OR = 5.26, 95% CI: 2.02-13.69). The prognosis of patients with CRC undergoing PALND was affected by some factors. For example, histologically poor differentiation was a risk factor for 3-year survival (HR = 2.98, 95% CI: 1.48–5.99) compared to that of medium-high differentiation. Patients with fewer than 4 PALN metastases had a survival benefit of 4 or more (HR = 1.88, 95% CI: 0.97-3.62). The presence of preoperative extra-retroperitoneal metastasis was significantly associated with poorer relapse-free survival (HR = 1.85, 95% CI: 1.10–3.10), while the absence of it was a protective factor of relapse-free survival. Age (HR = 1.00, 95% CI: 0.98–1.02), postoperative chemotherapy (HR = 1.01, 95% CI: 0.54–1.84), primary tumor location (HR = 0.93, 95% CI: 0.54–1.59), CEA level (<10 vs ≥10) (HR = 0.88, 95% CI: 0.41–1.86), and T grade (T4+3 vs T1-2) (HR = 1.35, 95% CI: 0.74–2.46) were not independent prognostic factors affecting survival (Table 2).

**Table 2:** Meta-analysis results of overall survival and hazard rates in further analysis

				Heterogeneity		
Pooled value	Studies	OR	HR	95% CI	*P* value	*I*^2^
Compare positive para-aortic lymph node group with negative group						
3-year OS	5	0.30		0.11–0.82	0.002	76%
5-year OS	4	0.19		0.11–0.31	0.31	16%
Compare complete resection group with not complete resection group						
3-year OS	5	5.26		2.02–13.69	0.14	42%
OS						
Histological differentiation (poor vs medium-high)						
HR	4		2.98	1.48–5.99	0.08	56%
Postoperative chemotherapy (yes vs no)						
HR	4		1.01	0.54–1.84	0.23	30%
Age (old vs young)						
HR	4		1.00	0.98–1.02	0.51	0
The number of PALN metastases(≥4 vs <4)						
HR	3		1.88	0.97–3.62	0.20	39%
Primary tumor location (colon vs rectum)						
HR	3		0.93	0.54–1.59	0.42	0
CEA (ng/ml)(<10 vs ≥10)						
HR	3		0.88	0.41–1.86	0.03	72%
T grade (T4+3 vs T1-2)						
HR	3		1.35	0.74–2.46	0.46	0
Disease-free survival						
Preoperative extra-retroperitoneal metastases (yes vs no)						
HR	4		1.85	1.10–3.10	0.75	0
Primary tumor location (colon vs rectum)						
HR	3		1.29	0.74–2.25	0.41	0

*OR* odds ratio, *CI* confidence interval, *OS* overall survival, *HR* hazard ratio

### Sensitivity analysis and publication bias

**Fig. 5:**
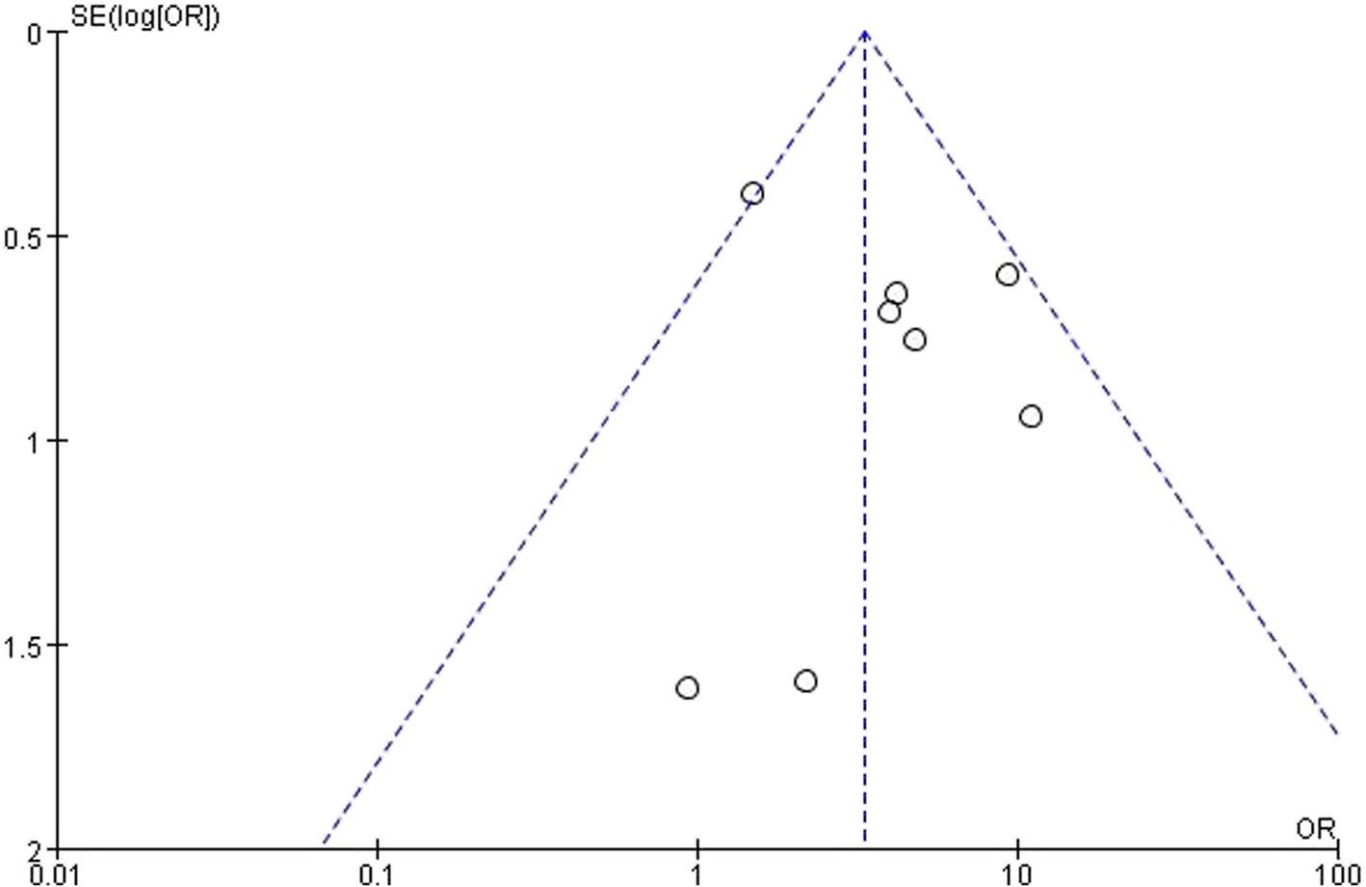
Funnel plot of 5-year overall survival between PALND group and control group. Meta-analysis comparing patients with colorectal cancer and clinically suspected PALN metastasis receiving PALND with those did not undergo PALND, and further analysis of the prognostic factors in PALND group

All outcome models were stable by sensitivity analysis, with the exception of a model that compared 3-year survival in the pathologically PALN-positive group and the pathologically PALN-negative group. After deleting Dumont's study [24], we found that the analysis model became more stable, which had little impact on the overall result. Therefore, we decided to keep this study. The heterogeneity may be related to the small sample size of Dumont's study. In the meta-analysis of 5-year OS between the PALND group and the control group, the *P* value of Begg’s test was 0.108 which meant the absence of reporting bias and the funnel plot was recorded in Fig. 5.

## Discussion

PALN metastases were defined as histologically confirmed retroperitoneal lymph nodes metastases rather than local tumor recurrence. The latter refers to tumor cell recurrence at a local site after surgical removal of the primary tumor, without lymph node involvement [24]. According to the classification system of the Japanese Society of Clinical Oncology, the retroperitoneal region, in which PALN metastases occur, is classified as A (supra-renal vessels) and B (infra-renal vessels) [28]. The upper boundary of the B region is the renal vein, and the lower boundary is the iliac bifurcation, surrounding the abdominal aorta and inferior vena cava. The A retroperitoneal area with a starting point slightly higher than the renal vein is unconventionally included in the anatomical range of PALN because of the difficulty of surgical resection and the low possibility of complete resection [2, 7].

Lymph nodes, a type of normal tissue structure, can be imaged and measured regardless of whether they are involved in metastasis. While biopsies are invasive and difficult to replicate, computed tomography, magnetic resonance imaging, and positron emission tomography are reproducible, straightforward, and practically noninvasive methods for measuring the size of lymph nodes. Although the literature included in this study must have clear radiological evidence supporting PALN metastasis as described above, the definition of measurable and identifiable target metastatic lymph nodes in terms of computed tomography is controversial. Some institutions measure the long axis diameter of lymph nodes to check for enlargement. For example, in the study by Sahara et al., the diameter of the long axis of lymph used lymph node long axis diameter ≥10 mm as the inclusion criterion [18]. Other groups have used the short axis for diameter measurement; however, they also contain varying lengths, such as 5, 8, and 10 mm [17, 19, 22, 29, 30]. There was still no consensus on the selection of lymph node diameter line, perhaps due to multiple medical equipment and different judgments of imaging technology scientists. Lymph node diameter measurement was also commonly used to evaluate the effect of chemotherapy or radiotherapy, namely the treatment response and post-resection recurrence by imaging. Therefore, patients with CRC should be routinely followed up every 3 months for the first 2 years and every 6 months thereafter [31].

There were two ways of time classification: one is the operative time of primary tumor and PALN mentioned in our study, and the other is the time of PALN metastasis after the diagnosis of primary tumor [11]. They should not be confused. According to our subgroup analysis results, the survival effect achieved by PALND was independent of the surgery time for primary tumor and clinically suspected PALN metastases. Increasing articles emphasized the classification of PALN metastasis time. Gagniere et al. manifested that the OS was not affected by PALN metastasis time (HR = 2.83, 95% CI: 0.61–13.08, *P* = 0.18) [2]. A retrospective analysis by Ichikawa et al. also showed that the 3-year RFS of 28 patients with PALND was not affected by the time of PALN metastasis (HR = 0.792, 95% CI: 0.66–3.58, *P* = 0.301) [23]. In another small sample study, Arimoto et al. reported that the 3-year OSs for simultaneous (*n* = 9) and metachronous (*n* = 5) PALN metastases were 40 and 100%, respectively [29]. Due to the small sample size and the inclusion of patients with other distant metastatic lesions, the conclusions of these three studies were not statistically significant. Choi et al. included 24 participants with pathologically positive PALN metastasis, but without other distant metastasis, indicating that patients with metachronous metastasis (*n* = 5) had a longer median survival time than patients with simultaneous metastasis (*n* = 19) (median OS: 61 months (95% CI: 50–71) and 29 months (95% CI: 1–57), *P* = 0.227) [11]. However, the results were not statistically significant. We were unable to analyze in detail whether there was a difference in survival between patients with simultaneous and metachronous PALN metastasis according to the time of PALN metastasis after the diagnosis of primary tumor because few studies have provided accurate time limits to distinguish these two groups of patients.

Radical surgical resection of stage I–III CRC is still the mainstay of treatment, which is associated with a 5-year OS ranging from 50 to 94% [32]. Although there have been several studies on retroperitoneal lymphadenectomy, the choice between PALND and adjuvant therapy remains uncertain. Some studies had reported no significant survival benefit from extensive lymphadenectomy [33, 34]. Based on the 20 studies we included, the 5-year OSs for patients with CRC undergoing PALND ranged from 0 to 70.3%, and the 3-year OSs ranged from 33.15 to 93.90%. Eight articles presented the 5-year OSs for participants who underwent PALND and those who did not, and pooled results suggested a survival benefit for patients who received PLAND (OR = 3.73, 95% CI: 2.05–6.78). Additionally, none of the patients included in these eight articles had other extra-retroperitoneal metastasis before surgery. Only 6/16 patients in the study by Ogura et al. and 3/13 in the study by Kim et al. did not confirm pathological PALN metastasis [12, 14]. To some extent, our analysis indicated that para-aortic lymphadenectomy rather than chemotherapy alone was beneficial to the survival of patients with CRC and clinically suspected PALN metastasis. The ideal margin of retroperitoneal lymph node resection should be negative, and our results were consistent (OR = 5.26, 95% CI: 2.02–13.69) (Table 2). Laparotomy and endoscopy are the alternative surgical approaches; however, few studies have compared their operative difficulty, duration, blood loss, and survival outcome. Furthermore, a recent case-cohort analysis found no difference in overall survival between endoscopic and open approaches (HR = 0.941, 95% CI: 0.571–1.831, *P* = 0.101) [10]. From the renal vessel to the iliac vessel bifurcation, PALND was conducted along the abdominal aorta. Because there are so many important vascular pathways nearby, PALND is a highly challenging surgical procedure and requires a more experienced general surgeon. The popularity of endoscopic surgery has undoubtedly increased the difficulty of lymph node dissection and also brought the risk of surgical complications to some extent. However, endoscopic approach had no significant survival benefit compared to open approach. Complications after PALND were reported in 18 included literatures, and the incidence of complications in the PALND group ranged from 8.00 to 42.90%. In terms of the number of complications, the most common complications in PALND group were intestinal obstruction (50 cases), followed by incision infection (44 cases), anastomotic leakage (28 cases), urinary retention (25 cases), pneumonia (21 cases), urinary tract infection (18 cases, abdominal abscess (18 cases), chylous leakage (16 cases), and abdominal hemorrhage (7 cases). Rare complications include atelectasis, venous embolism, ureter or bladder damage, and so on (Supplementary table 1). In addition, according to our result, PALND had no effect on the incidence of complications (OR = 0.97, 95% CI: 0.46–2.08).

Not all patients with suspicious preoperative imaging results of PALN have pathologically postoperative positive lymph nodes. After performing PALND on 33 patients with CRC who exhibited signs of PALN metastasis on preoperative radiologic examination, Lee et al. found that only 14 patients were confirmed as pathologically positive PALN metastasis [35]. In other words, the pathological findings of PALN metastasis had a 42.42 percent likelihood of agreeing with the radiological findings. The advantage of surgical resection over imaging detection is that it can provide comprehensive diagnostic pathological information to guide treatment in terms of genes. However, metastasectomy is not always feasible, especially if metastatic lymph nodes have invaded important blood vessels or organs, or if the patient's physical state prevents them from undergoing complex surgery. Except for metastasectomy, salvage adjuvant chemotherapy may be another option for patients with advanced CRC. Yeo et al. showed that radical chemotherapy is an effective salvage treatment for retroperitoneal lymph node metastasis in CRC, with a 5-year OS of approximately 36.4% [36]. Currently, National Comprehensive Cancer Network guidelines recommend the use of capecitabine-base and 5-fluorouracil-base as the most commonly used first-line chemotherapy regimens for CRC. If the disease progresses and distant metastasis to para-aortic lymph nodes occurs, targeted therapies may also be considered. Bevacizumab targeting vascular endothelial growth factor ligand and cetuximab targeting epidermal growth factor receptor are key standard agents for improving survival outcomes in patients with metastatic CRC [37].

Due to the small number of patients undergoing PALN metastasectomy and the limited literature on this topic, more prospective large multicenter randomized trials are urgently needed to confirm the merits of extended lymphadenectomy. However, the use of surgical resection, chemotherapy, or local radiation therapy can be incorporated into a multimodal strategy for the treatment of these patients.

## Conclusion

To sum up, PALND had a survival benefit for patients with CRC and clinically diagnosed para-aortic lymph node metastasis, in which pathologically negative PALN, complete resection, less metastatic PALN and medium-high histological differentiation were protective factors for survival and the presence of preoperative metastasis elsewhere was a risk factor for recurrence-free survival.

## Supplementary Information

**Additional file 1: Supplementary Table 1**. Rare complications include atelectasis, venous embolism, ureter or bladder damage, and so on.

## Data Availability

The datasets used and/or analyzed during the current study are available from the corresponding author on reasonable request.

## Abbreviations

CR: Complication rate
CRC: Colorectal cancer
HR: Hazard ratio
NOS: Newcastle–Ottawa scale
OR: Odds ratio
OS: Overall survival
PALN: Para-aortic lymph node
PALND: Para-aortic lymphadenectomy
R0: Complete resection
