# Supplementary material for: Survival benefits of para-aortic lymphadenectomy in colorectal cancer with clinically suspected para-aortic lymph node metastasis: a meta-analysis and systematic review
Source: World J Surg Oncol. 2023 Jan 31;21:28. doi: 10.1186/s12957-023-02908-y (PMC9887871; doi:10.1186/s12957-023-02908-y)
Supplement: Supplementary file 1 — Additional file 1: Supplementary Table 1. Rare complications include atelectasis, venous embolism, ureter or bladder damage, and so on. [file 12957_2023_2908_MOESM1_ESM.docx]

| Author,  Year | Morbidity | Ileus | Incision infection | [stomal leak](javascript:;) | [uroschesis](javascript:;) | [pneumonia](javascript:;) | [urinary tract infection](javascript:;) | [intra-abdominal abscess](javascript:;) | [chylous leakage](javascript:;) | [bleeding](javascript:;) | [pulmonary atelectasis](javascript:;) | [pulmonary embolism](javascript:;) | ureteral injury | Bladder leakage | Acute compartment syndrome | [deep venous thrombosis](javascript:;) | Enteritis/diarrhea | Delayed gastric emptying |
| --- | --- | --- | --- | --- | --- | --- | --- | --- | --- | --- | --- | --- | --- | --- | --- | --- | --- | --- |
| Gagnière,  2015 | 8% |  |  |  |  |  |  |  |  |  |  | 1 | 1 |  |  |  |  |  |
| Arimoto,  2015 | 50% | 1 |  | 1 | 2 |  |  |  |  | 1 |  |  |  |  |  |  | 1 |  |
| Choi,  2010 | 25.00% | 2 | 3 |  |  |  |  |  |  | 1 |  |  |  |  |  |  |  |  |
| Yamada,  2018 | 39% | 1 | 3 |  | 2 |  | 2 | 2 |  |  |  |  |  |  | 1 | 1 |  |  |
| Min,  2008 | 33% | 2 |  |  |  |  |  |  |  |  |  |  |  |  |  |  |  |  |
| Song,  2016 | 15% | 2 |  | 2 |  |  |  |  |  |  |  |  |  |  |  |  | 1 |  |
| Yamamoto,  2019 | 27% | 1 |  |  | 1 |  |  |  | 1 |  |  |  |  |  |  |  |  |  |
| Nakai,  2017 |  | 4 | 3 | 6 | 1 |  | 5 | 6 | 1 |  |  |  |  |  |  |  |  |  |
| Sakamoto,  2020 | 31% | 1 | 3 |  | 1 |  |  | 1 |  |  | 1 |  |  |  |  |  |  |  |
| Bae,  2016 | 7.90% | 3 | 4 |  | 3 |  |  |  |  |  |  |  |  |  |  |  |  |  |
| Lee.J.,  2021 | 18.20% | 18 | 6 | 11 | 13 | 1 |  | 5 |  | 2 |  |  |  |  |  |  |  |  |
| Liang,  2012 | 29.40% |  | 4 | 2 | 1 |  | 1 |  |  |  |  |  |  |  |  |  |  |  |
| Ichikawa,  2021 | 42.90% | 3 | 4 |  | 1 |  | 3 |  |  | 1 |  |  |  |  |  |  |  |  |
| Lee, S. C.,  2021 | 38.30% | 7 | 8 | 2 |  |  |  |  | 2 |  |  |  |  |  |  |  |  |  |
| Shibata,  2002 | 25% | 1 |  |  |  | 1 |  | 1 |  |  |  |  |  | 1 |  |  |  |  |
| Ogura,  2015 | 18.80% | 1 |  |  |  |  |  |  |  | 1 |  |  |  |  |  |  |  |  |
| Tentes,2007 | 17.74% |  | 3 | 1 |  | 2 |  | 2 |  |  |  |  |  |  |  |  |  |  |
| Sun,  2021 | 27.30% | 3 | 3 | 3 |  | 17 | 7 | 1 | 12 | 1 |  |  |  |  |  |  |  | 1 |
| total |  | 50 | 44 | 28 | 25 | 21 | 18 | 18 | 16 | 7 | 1 | 1 | 1 | 1 | 1 | 1 | 2 | 1 |
